# Multi-Odor Discrimination by Rat Sniffing for Potential Monitoring of Lung Cancer and Diabetes

**DOI:** 10.3390/s21113696

**Published:** 2021-05-26

**Authors:** Yunkwang Oh, Ohseok Kwon, Sun-Seek Min, Yong-Beom Shin, Min-Kyu Oh, Moonil Kim

**Affiliations:** 1 Bionanotechnology Research Center, Korea Research Institute of Bioscience and Biotechnology (KRIBB) 125 Gwahang-ro, Yuseong-gu, Daejeon 34141, Korea; oyk0213@kribb.re.kr (Y.O.); ybshin@kribb.re.kr (Y.-B.S.); 2 Department of Chemical and Biological Engineering, Korea University, 145 Anam-ro, Sungbuk-gu, Seoul 02841, Korea; 3 Infectious Disease Research Center, Korea Research Institute of Bioscience and Biotechnology (KRIBB) 125 Gwahang-ro, Yuseong-gu, Daejeon 34141, Korea; oskwon79@kribb.re.kr; 4 Department of Physiology and Biophysics, Eulji University School of Medicine, 77 Gyeryong-ro, Jung-gu, Daejeon 34824, Korea; ssmin@eulji.ac.kr; 5 KRIBB School, Korea University of Science and Technology (UST), 217 Gajeong-ro, Yuseong-gu, Daejeon 34113, Korea

**Keywords:** multi-odor discrimination, 2-choice/no-go, animal biosensor, olfactory behavior

## Abstract

The discrimination learning of multiple odors, in which multi-odor can be associated with different responses, is important for responding quickly and accurately to changes in the external environment. However, very few studies have been done on multi-odor discrimination by animal sniffing. Herein, we report a novel multi-odor discrimination system by detection rats based on the combination of 2-Choice and Go/No-Go (GNG) tasks into a single paradigm, in which the Go response of GNG was replaced by 2-Choice, for detection of toluene and acetone, which are odor indicators of lung cancer and diabetes, respectively. Three of six trained rats reached performance criterion, in 12 consecutive successful tests within a given set or over 12 sets with a success rate of over 90%. Through a total of 1300 tests, the trained animals (N = 3) showed multi-odor sensing performance with 88% accuracy, 87% sensitivity and 90% specificity. In addition, a dependence of behavior response time on odor concentrations under given concentration conditions was observed, suggesting that the system could be used for quantitative measurements. Furthermore, the animals’ multi-odor sensing performance has lasted for 45 days, indicating long-term stability of the learned multi-odor discrimination. These findings demonstrate that multi-odor discrimination can be achieved by rat sniffing, potentially providing insight into the rapid, accurate and cost-effective multi-odor monitoring in the lung cancer and diabetes.

## 1. Introduction

In general, animals instinctively detect situations that threaten them. Rodents, for example, use their strong sense of olfaction to recognize and avoid predators they have never seen before [1]. Some animals (i.e., dogs, rats, etc.) have superior olfactory discrimination abilities compared to humans. Such animals’ innate ability for odor detection and associative learning is the basis for animal nose biosensors [2,3]. Although understanding for odor recognition has not yet been fully clarified at the molecular level, animals conditioned with odor stimuli can quickly and accurately recognize VOCs (volatile organic compounds) and exhibit alerting behaviors as specific signaling behaviors [4]. Animal nose sensors, which utilize animal’s olfactory ability to detect specific odors, are gaining increasing interest in the fields of forensics and homeland security due to their cost efficiency and excellent detection performance [5,6], and has been mainly applied to drug detection [7,8] and mine/explosive detection [9,10]. In recent years, the animal nose sensors are expanding their applications to medical fields such as cancer detection [11,12] and blood sugar detection [13,14] with advantages of non-invasive detection and early detection of disease. In particular, the use of detection animals in biomedical applications, which is directly connected to human life and health, has an important meaning to complement the problems of existing device-based diagnostic methods. Rats or mice have several advantages as detection animals. Compared to canine, they eat small amounts of food, and are so small that a large number of rats can be handled by the experimenter. Therefore, maintenance is relatively economical [15,16]. Also, they are trained on small indoor apparatus. Therefore, reinforcement and punishment are given immediately after the animal’s behavior occurs, thus avoiding learning inefficiency due to the delay of reward timing [17]. In addition, many groups of detection rats or mice can be simultaneously trained by simply increasing the number of small and inexpensive training apparatus. Therefore, a sufficient amount of data can be acquired, leading to higher reliability by means of statistical analysis [18]. In this regard, rodents are the best candidates.

Multiple odors can be associated with different behavioral responses. Such a multi-odor discrimination is critical for responding quickly and correctly to environmental changes [19]. However, little studies have been done on multi-odor discrimination by animal olfactory detection. When it comes to odor discrimination learning, 2-Choice and Go/No-Go (GNG) tasks are most commonly used olfactory behavioral selection paradigms [20,21]. In the GNG task, animals are asked to go when a Go stimulus is shown, and wait when a No-Go stimulus is presented. In general, the 2-Choice involves a forced selection between the two responses based on the odor stimulus presented [20]. Another paradigm that seems to share many of the features of 2-Choice is GNG that involves a series of decisions in which animals are tasked with responding to one stimuli (the Go odorant stimuli) but not to another stimuli (the No-Go odorant stimuli) [21]. The GNG operation is basically the same as the 2-choice model in that there are two options. However, this task aims to respond to only one target odor. Therefore, the GNG paradigm is not suitable for multi-odor discrimination that identifies two or more target odors. In the current study, based on the advantages of the use of rodents as an odor detector, a novel animal nose sensor system for discriminating multiple odors was developed. This system was designed based on the combination of 2-Choice and Go/No-Go (GNG) into a single paradigm, in which the Go response of GNG was replaced by 2-Choice. The biggest drawback of GNG task is for animals to make more Go response [21,22]. In general, this phenomenon is triggered by GNG’s curious choice bias toward the Go response. The combined 2-Choice/No-Go paradigm has the advantage of reducing false alarms and increasing hit rates by removing the bias toward the Go response of GNG. In order to evaluate this system, in this study, toluene (C_6_H_5_CH_3_) and acetone (CH_3_COCH_3_) analytes were used as breath indicators for lung cancer and diabetes, respectively, based on previous clinical works [23,24]. Our system showed excellent performance for multi-odor discrimination through 1300 tests, and long-term stability of learned odor information.

## 2. Materials and Methods

### 2.1. Animals and Odors

Male rats (Wistar, over 4 weeks, Samtako Bio Korea, Osan, South Korea) were used for odor detection training. All animals were maintained under normal conditions with a 12-h light-dark cycle and were individually housed in transparent plastic cages with temperature and humidity adjusted to 25 °C and 40% relative humidity (RH). Prior to training, rats were food-restricted and maintained at approximately 85% of free-feeding body weight. Initial food restriction was accomplished gradually over 2 days prior to the first day of odor discrimination training. After training has been initiated, rats were fed once per day after the training and had free access to water throughout. During all training courses, each rats were systematically rewarded or punished according to their odor-sensing behavior. All data in the study was collected under the approved IACUS protocol (IACUC approval num-ber: EUIACUC 16-18). The odors used as target were toluene (C_6_H_5_CH_3_, purity > 99.8%, Sigma-Aldrich, MO) and acetone (CH_3_COCH_3,_ purity > 99.8%, Sigma-Aldrich, St. Louis, MO, USA). The target odors were used as a positive stimulant followed by rewards.

### 2.2. Spiked Breath Sampling

**Figure 1:**
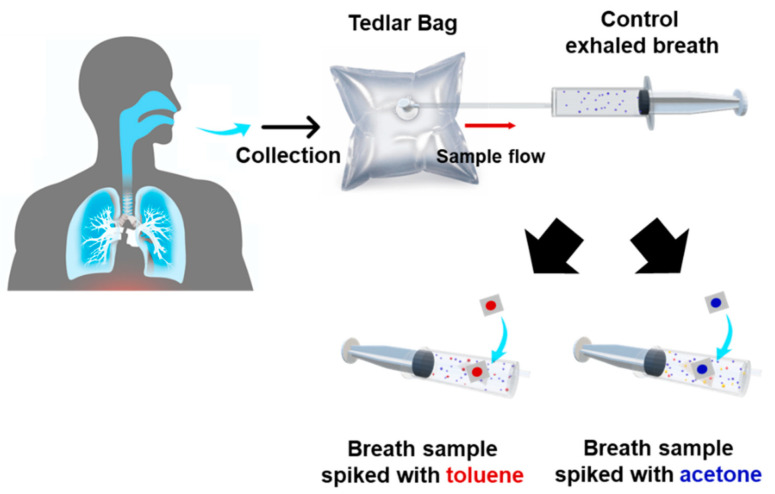
Schematic diagram of toluene- and acetone-spiked breath sampling. Human lung image captured form https://www.axetris.com, 12 March 2021 website.

Breath air samples were collected from healthy adult males and females (N = 4) aged 25–50 years. The procedure for collecting breath samples is as follows. Each exhalation provider holds the breath for 2 s and then exhales in the Tedlar bag. When the Tedlar bag is inflated by about 80%, the bag is locked by turning the stopcock attached to the bag so that the odor does not leak. As for spiked breath sampling (Figure 1), we followed the previously reported method [25].

### 2.3. Multi-Odor Discrimination Device

The multi-odor discrimination device is a cuboid shape made of acrylic that measures 60 × 63 × 45 mm (length × width × height). The device is divided into two upper and lower chambers, odor discrimination chamber and odor injection chamber. There is an odor injection hole in the middle of the bottom of the upper chamber, and this hole is connected to the odor delivery tube (3 mm in inner diameter) in the odor injection chamber. The tube is connected to three syringes to provide the targets or control. A ventilation fan (100 mm in size) is installed on the back wall of the odor discrimination chamber connected to the duct hose to discharge the odor inside the device to the outside of the laboratory. There are a couple of ledges, black and white ledges, installed inside both walls of the odor discrimination chamber. Food holes (15 mm in diameter) for serving food pellets are formed 35 mm above the floating ledges.

### 2.4. Performance Measurement

Data analysis in odor discrimination performance was calculated by the following standard formulas, including the following accuracy, sensitivity and specificity (Table 1).

## 3. Results and Discussion

### 3.1. Multi-Odor Discrimination Device Based on the Combined 2-Choice/No-Go Paradigm

Multiple odors can be associated with different behavioral responses. Such a multi-odor discrimination is critical for rapid and accurate responses to environmental changes. More than anything else, in multi-odor discrimination, the amount of limited valuable sample can be conserved, and time and costs required for odor sensing can be reduced. In general, the Go/No-Go (GNG), which is one of the most commonly used one of the existing odor discrimination tasks, is not appropriate for multi-odor discrimination learning, since it aims to respond to only one target odor. In addition, the GNG task has a critical drawback such as curious bias for the Go responses, resulting in the increased error rates. Therefore, we wished to design a multi-odor discrimination device with a new selection paradigm to overcome the shortcoming of the GNG. The system was designed based on the combined 2-Choice/No-Go paradigm in which GNG’s Go response was replaced by 2-Choice. The operation principle of this system is that 2-Choice task and No-Go task act as target behaviors in response to target odors (i.e., toluene and acetone in this study) and control, respectively, thereby enabling discrimination learning for three odors including control.

**Figure 2:**
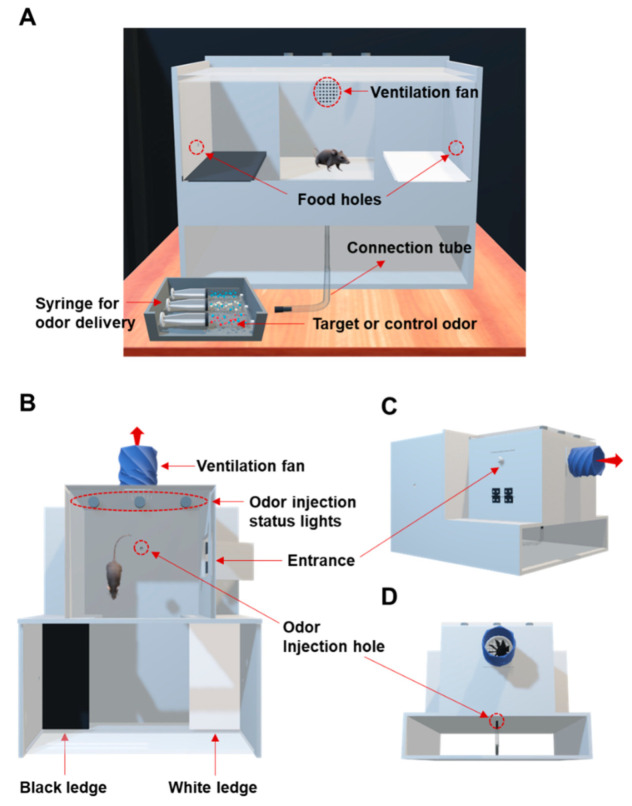
Schematic diagram of the multi-odor discrimination device based on the combined 2-Choice/No-Go paradigm. (**A**) 3D-front view, (**B**) 3D-top view, (**C**) Lateral-view of the backward and (**D**) Rear-view of the apparatus.

Figure 2 represents schematic diagram showing the layout of the multi-odor discrimination system. The system consists of upper and lower chambers. The upper chamber is the multi-odor discrimination unit, and the lower chamber is the odor injection unit. The odor injection unit has an odor tube and syringes for odor injection. A certain amount of odor can flow into the odor discrimination chamber through the odor hole connected to the odor tube attached to the syringe. The target odors are prepared with spike samples in which the disease-related odors are mixed with the exhaled breath, while the control odor is prepared with unspiked breath. The animal sniffs through the odor hole and decides whether to perform alerting behaviors. There are black and white floating ledges, which are structures for sign-tracking responses, on both sides of the walls of the odor discrimination chamber. Animals are followed by behavioral rewards and punishments depending on the outcome. 

**Figure 3:**
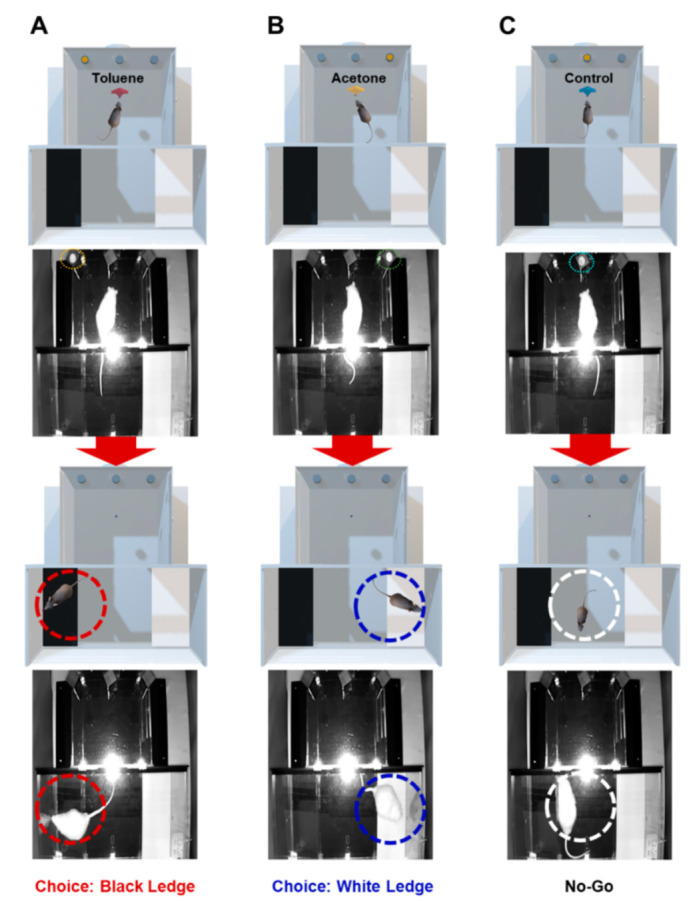
Photograph of the multi-odor discrimination device. (**A**) Choice of black ledge in response to toluene. (**B**) Choice of white ledge in response to acetone. (**C**) No-Go in response to control.

As shown in Figure 3, when an animal recognizes toluene and acetone, it jumps onto the black and white ledges to receive food rewards, respectively. When control is provided, the animal’s target behavior is to remain in the odor discrimination chamber without choosing a ledge. Choosing the No-Go in response to target odors or jumping onto a ledge in response to control is considered a false behavior. In this case, the punisher is provided with an unpleasant stimulus such as noise or stick bend-in immediately after the undesired behavior in order to reduce the frequency of error responses. A ventilation fan is installed on the wall behind the odor discrimination chamber. The odor remaining inside during each test can be discharged to the outside through the operation of the ventilation fan.

### 3.2. Animal Training for Multi-Odor Discrimination

**Figure 4:**
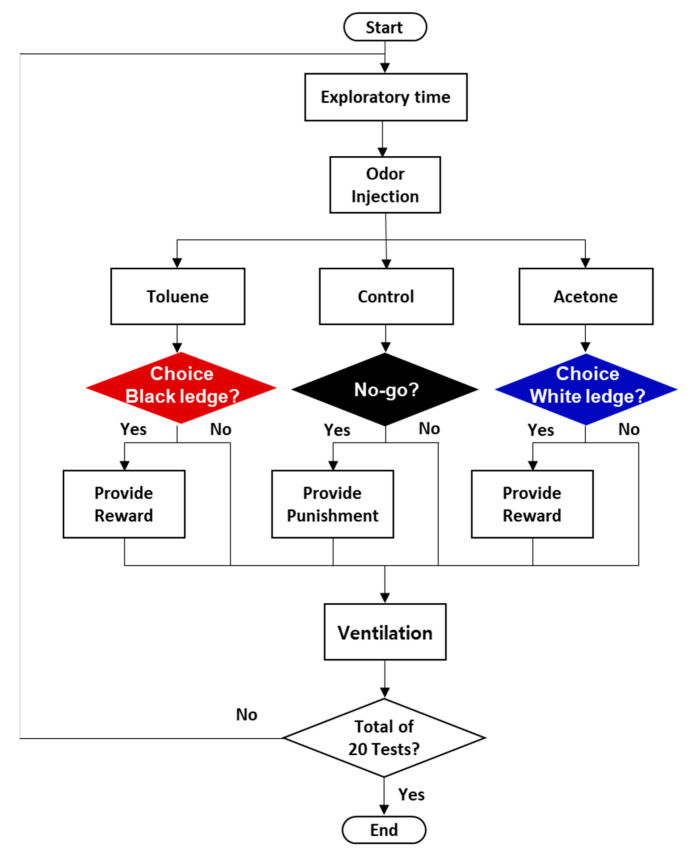
Flow chart for the multi-odor discrimination based on the combined 2-Choice/No-Go paradigm.

Figure 4 shows a flow chart for multi-odor discrimination. The training to discriminate multiple odors was established based on the combined 2-Choice/No-Go paradigm. Animal multiple odor discrimination device consists of six steps. The first step is the beginning part of training to place rats in the odor discrimination chamber. Rats can freely explore in the chamber before the start of training until they calm down, so that they are ready to start discrimination training. The second step is to inject the odors to the chamber. When the rat approaches the odor hole, the target or control odors flow in the chamber. In the third step, the rat decides which task to choose after recognizing the odor. The animal can choose 2-Choice task or No-Go task in response to the odor. The fourth step is to judge the occurrence of desirable or undesirable behaviors. The desirable behavior is for the animal to jump on the black and white ledges in the presence of toluene and acetone, respectively, or to remain still in response to control odor, while the undesirable behavior is to choose the No-Go task when toluene and acetone are provided, or to jump to the ledges in response to control. If the animal fails to jump onto the ledges within 10 s after the target odors are provided, it will be considered an error response and punished, regardless of the outcome of the signal behavior. Reinforcer or punisher are offered according to the outcome of the behavior in the fifth step. Food rewards are provided to motivate desirable behavior, and penalties are provided to reduce the frequency of undesirable behavior. When a target behavior occurs following the injected odor, a click sound is immediately made using a clicker, which is to bridge the time gap between behavior and reward. The sixth step is the last part of training in which the inside of the chamber is completely ventilated for 40 s through a ventilation fan for the next test. All steps from the first to the sixth step are performed continuously. Each test takes about 50–60 s including ventilation, and one set of 20 tests takes 17–20 min. To minimize false responses, all tests in this study were blind tests. Also, the syringes were replaced with new ones for each set, and after a series of training, the odor tubes were thoroughly cleaned and deodorized to prevent cross-contamination of odors, since odor contamination can lead to false responses. Exhaled breath samples used in this study were collected by the method described in the Materials and Methods section.

### 3.3. Measurement of Multi-Odor Detection Performance

**Figure 5:**
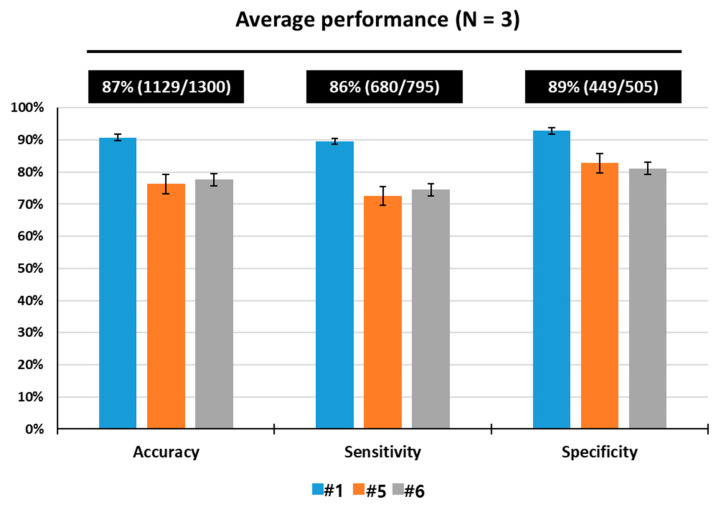
Discrimination performance of rats (Number 1, 5 and 6) in response to toluene and acetone in spiked breath through a total of 1300 tests.

To assess the multi-odor discrimination performance, three rats (numbers 1, 5 and 6) were trained using breath samples spiked with toluene, a lung cancer-related odor indicator, and acetone, a diabetes-related odor indicator. All of three trained rats reached performance criterion, 12 consecutive successful tests within a given set or over 12 sets with a success rate of over 90%, in acquisition of the identification of three odors. On average, the rats (N = 3) required 43 sets training (=860 tests) to learn to make odor-reward association. As shown in Figure 5, the rats showed excellent performance for multi-odor discrimination by identifying the toluene- and acetone-spiked samples with 88% accuracy, 87 sensitivity and 90% specificity through a total of 1300 tests. Table 2 shows one-way ANOVA comparisons exhibiting detection performance of the rats for multiple odors. These results indicate that the rats were efficiently trained in our training device to acquire the multi-odor discrimination. 

Spiked breath samples contain a variety of non-target odorants. We wished to examine whether the trained rats were able to discriminate multiple odors in the presence of strong disturbing odor components contained in the exhaled samples. For this, a rigorous test was attempted using garlic and onion odors. It was observed that the rats were able to appropriately discriminate multiple odors even in the presence of interfering substances in the exhaled breath, with similar performance as in non-rigid tests (data not shown). Taking association of smoking with volatile organic compounds (VOCs) in exhaled breath, the VOCs might reflect the cumulative smoking exposure and accumulated risk that will finally lead to the initiation of lung cancer. In this regard, further study is thus required to examine any difference when tests were performed using breath samples from smokers and nonsmokers, and the results will be reported in next studies.

Rats can successfully learn multiple stimulus-response associations after repetition training for a certain number of courses when the outcomes are predicted [26,27]. Modification in the neural circuit networks during such odor discrimination learning closely related to decision making are not well elucidated. Millman and Murthy reported that some olfactory areas such as olfactory tubercle and posterior piriform cortex are responsible for predicting the rewards from odor input and relaying this information to behavioral responses [28]. Recently, Najafi et al. reported that as multi-sensory stimuli discrimination learning progressed, the activity of brain neural networks and neurons (i.e., excitatory and inhibitory neurons) related to decision making in the rodent’s brain increased significantly [29]. In that study, it was observed that more neurons were involved in learning and responded more quickly as rodents became proficient in trained discrimination tasks, which denotes that the neural network of the brain becomes more selective and focused. In this regard, the number 5 and 6 rats, which showed relatively poor performance for multi-odor discrimination, may possess a neural circuit pattern responding less selectively and indiscriminately in the presence of target odors. On the other hand, it seems possible that the number 1 rat may display a specific and clear neural network in response to the target odors. The neural networks and neurons of the animal with excellent performance for discriminating multiple odors may be well-prepared before the target behaviors occur. In this aspect, it is assumed that the multi-odor discrimination learning process is equivalent to a process of selectively patterning the neural network to specific odor stimuli.

### 3.4. Response Time

**Figure 6:**
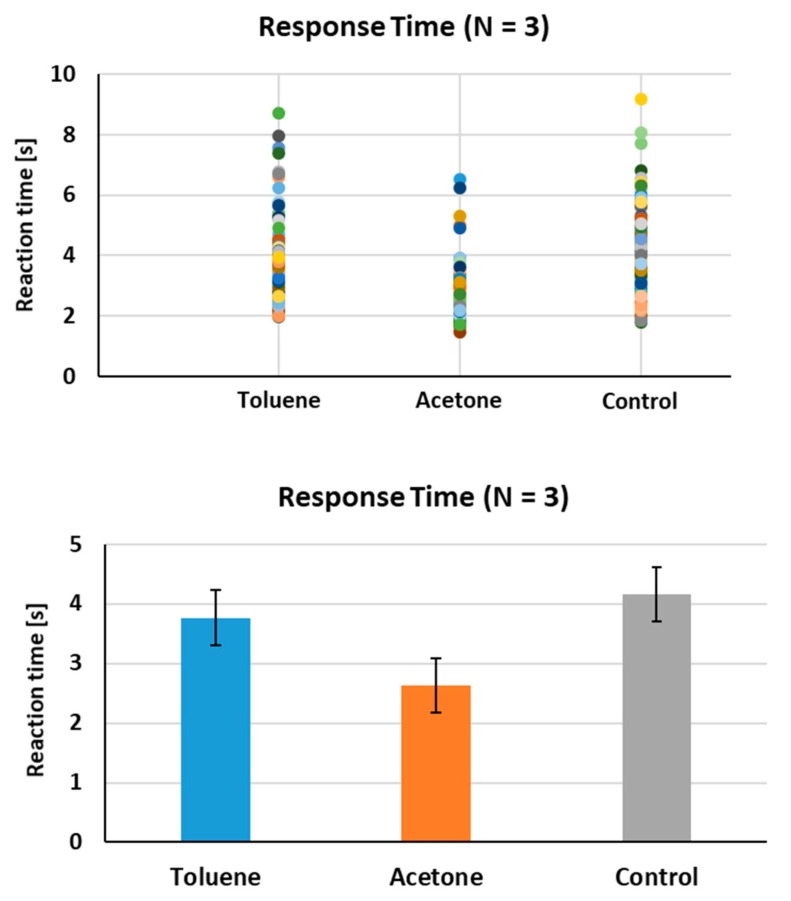
Animal response time to odors. The response time in discriminating both targets (toluene and acetone) and control at increasing concentrations was compared (**Upper panel**). The average response time for all of the tested rats (Number 1, 5 and 6) is shown (**Lower panel**).

Each of the tests is completed every 50–60 s on average in rats under our experimental settings. The set is configured in 20 tests. So, the time required for the acquired set is about 17–20 min. The test time performed consists of odor presentation and response time, reward or punishment delivery time and ventilation time. The most important factor in determining the deviation of test time is response time. The response time was defined as the time taken for animals to jump onto the ledge after target odor presentation or to stop moving after control odor presentation. The purpose of this study is to discriminate different odor components from animal sniffing. There are studies showing that the detection performance of a living animal-based biosensor has a correlation with the response time [30,31,32]. Therefore, we wished to verify whether the two target odor components display a difference in the response time, or whether the response time of the target odor was different compared to the control breath samples. As shown in Figure 6, the average response time of the three tested rats to toluene was about 3.76 s, which was longer than that to acetone (about 2.63 s). When the sensitivity was individually expressed as toluene and acetone, toluene and acetone sensitivities were about 87% and 86%, respectively. This result indicates only a weak or no correlation between sensitivity and response time in our experimental settings. The difference in response time between the two odors can rely on the odor preference between animals, the chemical structure of the odor molecules, the type of reinforcement, etc. A further study is necessary for the causes of this phenomenon. In addition, it was observed that the response time for both target odors was longer than that of the control breath (about 4.16 s, Figure 6). In general, when the performance accuracy is at chance levels (i.e., in initial trials of a new discrimination task or in acquiring a particularly difficult task), the response times for both target and control odors are essentially the same. In this study, the animals had tendency to spend more time in recognizing the control odor, while less time in identifying the target odors. This result is in a good agreement with previously reported results on the relationship between response time and accuracy [30]. This phenomenon can provide an indirect sensitivity index of odor discrimination by animal sniffing, as the accuracy increases, the response time to the target odors decreases.

### 3.5. Quantitative Analysis

**Figure 7:**
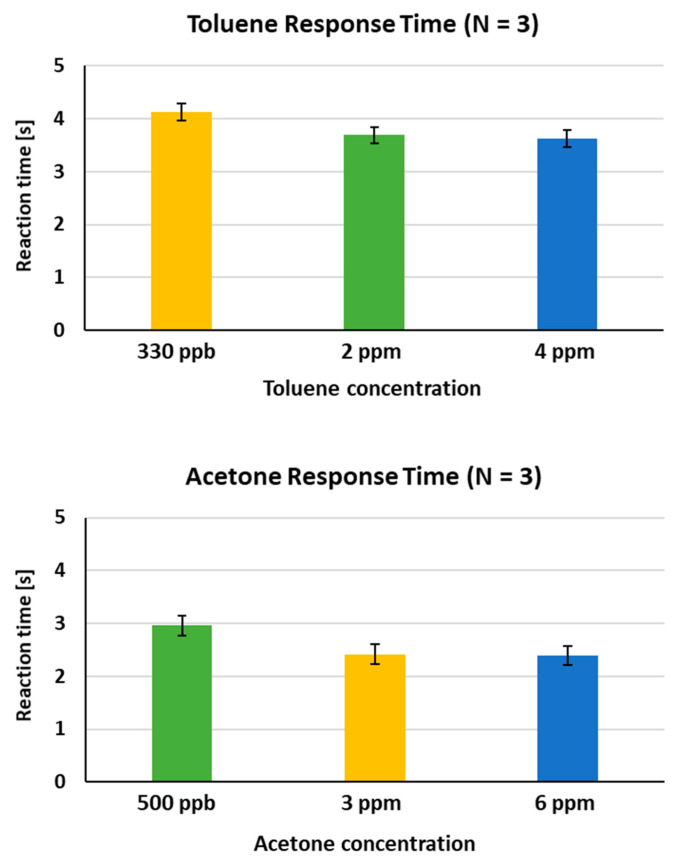
Quantitative measurement. The response time of the trained rats in discriminating toluene or acetone at increasing concentrations was compared (**Upper panel**). The average response time for all of tested rats (Number 1, 5 and 6) is shown (**Lower panel**).

In case of analysis of information on human health like disease diagnosis by specific odors, the determination of the amount of disease markers is very important in terms of early diagnosis and risk prediction of disease. In animal nose sensors, changes in behavior in response to odors are monitored as detection signals. So, it is impossible or very difficult to measure the animal’s behavioral changes quantitatively. For the reason, we wished to examine whether the behavioral signals exhibited by the detection rats could provide a quantitative measurement of multi-odor discrimination. For this, during the course of tests, the time taken for rats to jump onto the ledge after odor presentation was measured. As shown in Figure 7, the average response time for the three tested animals was observed to be about 4.13 s, 3.69 s and 3.63 s in response to toluene at increasing concentrations (330 ppb, 2 ppm and 4 ppm), and about 2.96 s, 2.41 s and 2.39 s in response to acetone at increasing concentrations (500 ppb, 3 ppm and 6 ppm), respectively. This result shows a dependence of behavior response time on odor concentrations under given concentration conditions, indicating that the trained rats could potentially perform quantitative measurement. The exhaled toluene level in lung cancer patients is known to be approximately 80–100 ppb [23], while the acetone concentration in the exhaled breath of diabetes patients is typically above 1.8 ppm [24]. The level obtained from this multi-odor discrimination study is approximately 3.3-fold higher and 3.6-fold lower than that observed in the breath of early lung cancer and diabetes patients, respectively. Thus, the sniffer rats’ sensitivity to toluene may need to be improved for clinical significance.

### 3.6. Measurement of Long-Term Retention

The long-term stability of learned olfactory memory is critical for practical application of animal nose sensors. In the case of a device-based sensor, the accelerated aging test, which helps determine the long-term stability under accelerated aging conditions within a shorter time, can be used to alleviate the time burden required for a long-term retention test. However, live animal-based sensors cannot be tested under the under accelerated aging conditions due to animal cruelty issue. Thus, long-term retention of learned information in animals has not been well explored. We attempted to measure the multi-odor discrimination performance of rats after a 45-day rest period. The tested rats stayed in the cages for the given rest period without additional training. 

**Figure 8:**
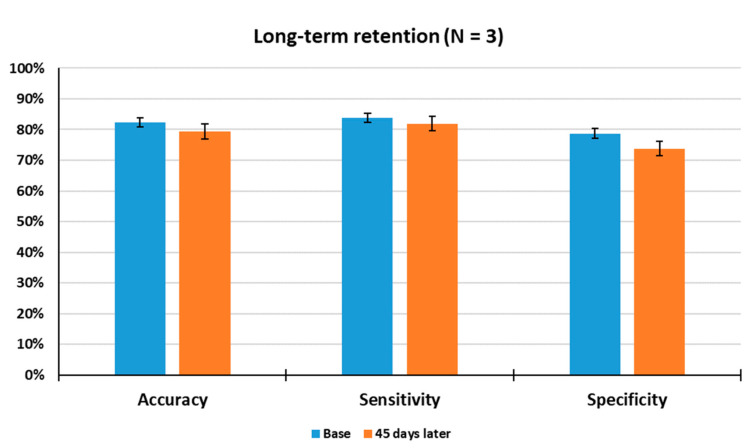
Measurement of long-term retention. The trained rats (Number 1, 5 and 6) were tested 45 days after the last training to gain insight into the long-term retention of the learned multi-odor discrimination. Base: Average test results for 1 week before long-term retention test.

As shown in Figure 8, the average accuracy, sensitivity and specificity were observed to be 80%, 82% and 76% through a total of 140 tests, respectively, when tested after a 45-day rest period. The results denote that long-term stability of learned multi-odor discrimination appears to be unimpaired in all of the tested rats. In particular, the performance of all rats measured under a given condition was within the range of ±10% deviation, suggesting that the trained rats well maintained the learned multi-odor information over a long period of time.

Each olfactory neuron has one type of olfactory receptor, which interacts with its specific odor molecule. Olfactory neurons are known to be constantly regenerated from progenitor cells or stem cells constituting the olfactory epithelium, and are regenerated every about 40–50 days [33]. Also, the neural networks and neurons in the brain are modified when animals associate specific odor stimuli with their cognate responses under the reward prediction. The examples of these modification include the increased number of neurons and higher levels in olfactory receptor expression. When the hippocampus is continuously stimulated through repetitive learning for odor discrimination, the hippocampus regards this odor stimuli as important information. The odor information stored in the hippocampus is relayed to the olfactory cortex, so that the learned odor discrimination information remains robust. Long-term retention of learned odor discrimination can be partly explained by this. Long-term storage of odor information is related to preference for odorant substances and emotions or experiences related to the odor, so a multilateral understanding of the mechanisms underlying long-term storage is needed.

## 4. Conclusions

In order for animal nose sensors to respond correctly to environmental changes regarding odor emissions, various odor stimuli need to be associated with their cognate response. So far, the substantial studies of multi-odor discrimination learning has not been well explored. Here, we report an animal nose sensor system for discriminating multiple odors. In order to minimize the bias toward the Go response of the Go/No-Go (GNG) task and maximize odor discrimination efficiency, the Go task in GNG was substituted for 2-choice task in the present system. This system, designed based on the combined 2-Choice/No-Go paradigm, uses the No-Go task in response to control odor and the 2-Choice task in response to target odors. Our multi-odor discrimination system distinguished multiple odors with high accuracy, sensitivity, and specificity using rats’ innate ability for odor detection and associative learning. We confirmed the reliability and robustness of this system by testing lung cancer-related and diabetes-related odor indicators. A dependence between the concentration levels of multiple odors and the response time of the animals was observed, implying that multiple odors could be quantitatively measured using the system. In addition, the multi-odor discrimination learned was maintained for 45 days, verifying long-term stability. These results suggest that animal nose sensor systems could be a useful tool for multi-odor screening for diseases with gaseous biomarkers in non-invasive manner. Despite the potential usefulness of this system for multi-odor discrimination learning, the test setup presented in this study is not in optimal conditions. Therefore, optimization of conditions such as number of tests, duration of test, and intervals between sets may be necessary. For a deeper understanding of the neural circuit mechanism for multi-odor discrimination learning in animals, it may be necessary to introduce optogenetics, fMRI, or integration of fMRI with optogenetic manipulation into the multi-odor discrimination system.

## Figures and Tables

**Table 1:** Data presentation in odor discrimination performance.

		Presence of Target Odor	
		Yes	No
Animal tests	Positive	a	b
	Negative	c	d

(1) Accuracy = (a + d)/(a + b + c + d) × 100 (%). (2) Sensitivity = a/(a + c) × 100 (%). (3) Specificity = d/(b + d) × 100 (%).

**Table 2:** Detection performance. One-way ANOVA comparisons (Accuracy: *p*-value < 0.001; Sensitivity: *p*-value < 0.001; Specificity: *p*-value < 0.01).

	Detection Performance		
	Number 1	Number 5	Number 6
Mean Accuracy (MA ± standard error S.E.)	91.0% ± 0.2	82.3% ± 0.5	83.7% ± 0.4
Mean Sensitivity (MS ± standard error S.E.)	89.6% ± 0.2	80.7% ± 0.5	86.3% ± 0.5
Mean Specificity (MS ± standard error S.E.)	93.2% ± 0.3	85.6% ± 0.7	81.5% ± 0.6

## Data Availability

The data presented in this study are available on request from the corresponding author (M.K.).
